# Velocity Control of Traveling-Wave Ultrasonic Motors Based on Stator Vibration Amplitude

**DOI:** 10.3390/s19235326

**Published:** 2019-12-03

**Authors:** Zhiwei Fang, Tianyue Yang, Yuanfei Zhu, Shiyang Li, Ming Yang

**Affiliations:** Department of Instrument Science and Engineering, Shanghai Jiao Tong University, Shanghai 200240, China; sjtufzw@sjtu.edu.cn (Z.F.); Thomas-yang@sjtu.edu.cn (T.Y.); zhuyuanfei@sjtu.edu.cn (Y.Z.); shiyangli@sjtu.edu.cn (S.L.)

**Keywords:** traveling-wave ultrasonic motors, stator vibration amplitude, parallel resonance frequency, velocity control

## Abstract

Nonlinearity and resonance frequency shift make it difficult to control the operation of the traveling-wave ultrasonic motors (TWUSMs) in a wide velocity and load range. In this paper, a velocity control scheme based on the stator vibration amplitude and the parallel resonance frequency (VCBVF) of TWUSMs is proposed. Then, the stator vibration amplitude (SVA) and parallel resonance frequency ($f_{p}$) are detected by a transformer ratio-arm bridge. Based on the linear relationship between the velocity and the SVA of TWUSMs, the proposed scheme achieves the control of the mechanical loop and the electrical loop. The linear relationship between the velocity and the SVA makes the mechanical loop achieve the target velocity efficiently, according to the SVA, and the electrical loop could provide the target SVA quickly. Experimental results show that the response time of velocity is 3–4 ms under different load torques and the overshoot is less than 22%. In addition, the proposed scheme improves the efficiency of TWUSMs due to $f_{p}$ tracking. Due to directing the SVA control, the proposed scheme can heighten the velocity response and the load adaptability of TWUSMs, and promote the application of TWUSMs under various conditions.

## 1. Introduction

Traveling-wave ultrasonic motors (TWUSMs) are widely used in microrobots, aerospace, and medical instruments due to its numerous advantages, such as quietness, no electromagnetic interference, low speed in high torque, and fast dynamics [1,2,3,4,5]. In some applications, such as the robotic system, TWUSMs need to operate under a wide range of velocity and load. However, the traditional methods cannot achieve excellent control effects due to the nonlinearity and the resonant frequency drift of TWUSMs. Therefore, it is crucial to propose a higher performance control scheme.

A TWUSM is driven by the two-phase sinusoidal waves, which excite elliptical motion on the stator [6,7]. Generally, the velocity of TWUSMs is controlled by the voltage amplitude [8], the driving frequency [9,10,11,12,13], or the phase difference between the two-phase sinusoidal waves [14]. However, the resonance frequency may drift significantly when the operating conditions change [1]. For the phase difference and the voltage adjustment methods, frequency drift could degrade the output characteristics. The driving frequency-based velocity control scheme (FBVC) could adapt to the resonance frequency variation. Some researchers use a voltage-controlled oscillator (VCO) to adjust the driving frequency to control the velocity of the TWUSM [9]. However, the nonlinear relationship between the velocity and the driving frequency limits the performance of the controller. In order to solve this problem, different control algorithms, such as fuzzy logic control [10], intelligent proportional-integral-derivative (PID) [11], nonlinear Hammerstein model [12], and a two-input sliding mode control algorithm [13] are proposed. Although these methods can compensate the nonlinearity, it is difficult for the microcontroller to implement the complex algorithms. In addition, authors in Reference [15] find that TWUSMs operating at the parallel resonance frequency ($f_{p}$) can maintain the high stability and low power consumption. However, adjusting the driving frequency could cause TWUSMs to operate far away from $f_{p}$ in most conditions. Actually, the output characteristics of TWUSMs are directly related to the stator vibration amplitude (SVA) [16,17,18]. Therefore, some researchers use the feedback electrode to detect the SVA and adjust the driving frequency to control the SVA to roughly achieve the target velocity of TWUSMs under constant load conditions [9]. However, the accurate and fast control of the velocity under various load conditions remains a challenge.

In order to improve the dynamic performance and efficiency of TWUSMs under various conditions, a velocity control scheme based on the stator vibration amplitude and the parallel resonance frequency (VCBVF) of TWUSMs is proposed. First, we analyze the relationship between the velocity and the SVA of the TWUSM. Second, a method for detecting the SVA and $f_{p}$ by a transformer ratio-arm bridge without the feedback electrode is proposed in our research. Then, the control scheme that contains the mechanical loop and the electrical loop is realized. Finally, the proposed scheme is verified and compared with the traditional scheme in terms of the velocity response, load adaptability, and efficiency.

## 2. Theoretical Analysis

### 2.1. Relationship between Velocity and Stator Vibration Amplitude

The TWSUM is mainly composed of a stator, a rotor, and piezoelectric elements. The piezoelectric elements are bonded to the stator and a friction liner is affixed to the rotor. The piezoelectric elements are excited by the two-phase sinusoidal waves with a $90^{{}^{\circ}}$ phase shift, producing a traveling wave on the stator. Then, the vibration of the stator is converted into the rotary motion of the rotor under the action of the axial pressure. When the load is constant, the rotor velocity can be described by the equation below [19].

(1) $$\Omega =-\gamma \frac{60hf}{r^{2}}A$$

where Ω is the rotor velocity with a unit of rpm, A denotes the SVA, f is the working frequency of the TWUSM, h is the offset distance from the centerline, r is the radius of the rotor, and γ is the velocity coefficient. For the TWUSM, h and r is constant. γ remains constant when the load is constant. Moreover, the velocity is linear to the SVA in the TWUSM due to the small variation of the working frequency.

### 2.2. Detection of $f_{p}$ and Stator Vibration Amplitude

**Figure 1:**
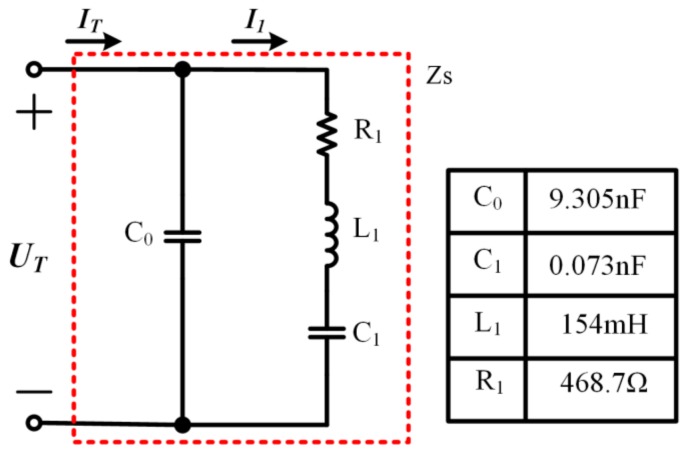
BVD equivalent circuit model of a single-phase piezoelectric element.

A single-phase piezoelectric element of a TWUSM is expressed by a Butterworth-Van Dyke (BVD) equivalent circuit model [20], as shown in Figure 1. The model contains a mechanical arm and an electric arm. The mechanical arm is composed of a motional resistance $R_{1}$, a motional inductance $L_{1},$ and a motional capacitance $C_{1}$. Meanwhile, the electric arm has a static capacitance $C_{0}$.

In the model, the stator vibration velocity v is proportional to the motional current $I_{1}$ [21].

(2) $$v\propto I_{1}$$

**Figure 2:**
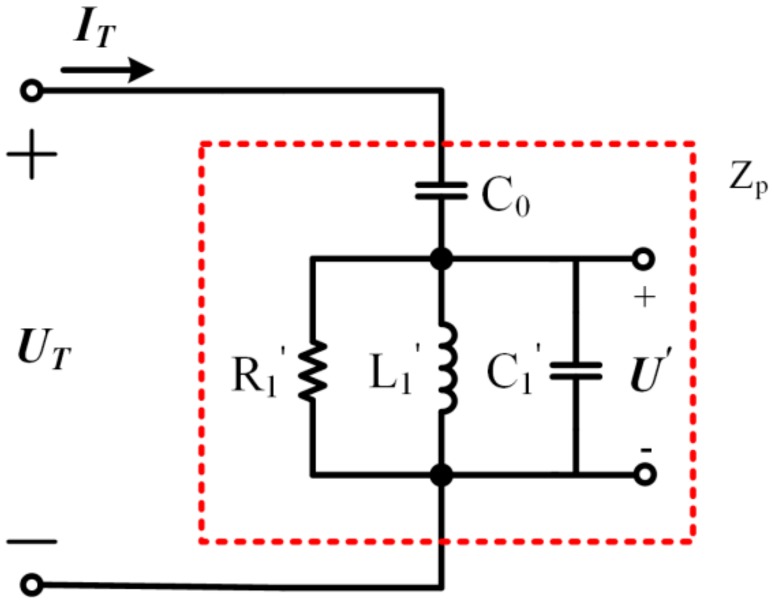
Transformation of the BVD equivalent circuit model.

In order to detect the SVA and $f_{p}$, the BVD equivalent circuit model can be transformed, as shown in Figure 2 [22].

Where $R_{1}^{\prime }$, $L_{1}^{\prime },$ and $C_{1}^{\prime }$ can be deduced as the following formulas.

(3) $$\left\{\begin{array}{cc} R_{1}^{\prime }= & \frac{1}{{\left(2\pi f\right)}^{2}C_{0}^{2}R_{1}}\\ L_{1}^{\prime }= & \frac{C_{0}C_{1}/\left(C_{0}+C_{1}\right)}{{\left(2\pi f\right)}^{2}C_{0}^{2}}\\ C_{1}^{\prime }= & {\left(2\pi f\right)}^{2}C_{0}^{2}L_{1} \end{array}\right.$$

Therefore, $L_{1}^{\prime }$ and $C_{1}^{\prime }$ resonate at $f_{p}$, which can be expressed as the formula below.

(4) $$f_{p}=\frac{1}{2\pi \sqrt{L_{1}C_{0}C_{1}/\left(C_{0}+C_{1}\right)}}=\frac{1}{2\pi \sqrt{L_{1}^{\prime }C_{1}^{\prime }}}$$

It can be inferred that the phase difference θ between the partial voltage $U^{\prime }$ and the input current $I_{T}$ changes with the driving frequency and it reaches 0° at $f_{p}$. According to Figure 1 and Figure 2, we can deduce that the following is true.

(5) $$\left\{\begin{array}{l} U_{T}\cdot j2\pi fC_{0}+I_{1}=I_{T}\\ \frac{I_{T}}{j2\pi fC_{0}}+U^{\prime }=U_{T} \end{array}\right.$$

where $U_{T}$ is the voltage applied to the piezoelectric element. Therefore, we can deduce that the following formula is true.

(6) $$I_{1}=-j2\pi fC_{0}U^{\prime }$$

The relationship between the stator vibration velocity v and the stator vibration amplitude A can be expressed by the following Equation.

(7) v=j2πfA

In this case, $I_{1}$, $U^{\prime }$, v, and A are vectors written in bold letters and j is the imaginary unit. According to Equation (2), Equation (6), and Equation (7), A can be expressed by the following formula.

(8) $$A\propto -2\pi C_{0}U^{\prime }$$

Therefore, the SVA can be characterized by the partial voltage $U^{\prime }$.

## 3. Implementation of the Proposed Scheme

### 3.1. Hardware Structure

**Figure 3:**
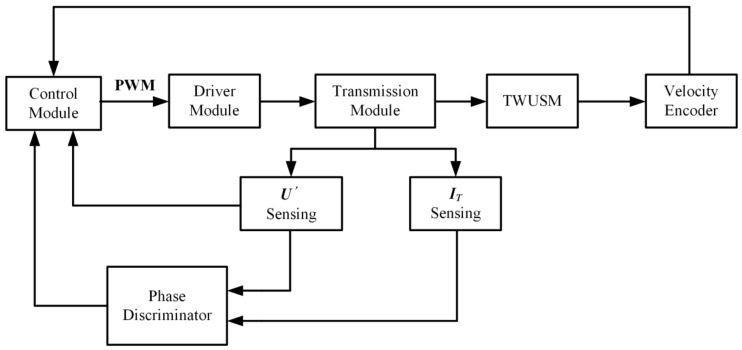
Schematic diagram of the TWUSM control system.

Figure 3 shows a schematic diagram of the TWUSM control system based on the proposed scheme. The transmission module transmits the excitation voltage to the TWUSM while detecting $U^{\prime }$ and $I_{T}$. Then, the system obtains the phase difference θ between $U^{\prime }$ and $I_{T}$ through the phase discriminator. In addition, a velocity encoder (1000 lines resolution) is used to detect the velocity of the TWUSM. The sampling interval of the velocity Δt depends on the velocity and the encoder resolution and Δt can be described by the following Equation.

(9) $$\Delta t=\frac{60}{\Omega \cdot N}$$

where Ω is the rotor velocity of the TWUSM with a unit of rpm and N is the encoder resolution. The control module detects the SVA, according to $U^{\prime },$ and detects $f_{p},$ according to θ, and generates pulse width modulation (PWM) waves. Then, a switching circuit is applied to convert direct current (DC) voltage into excitation voltage, according to PWM waves.

**Figure 4:**
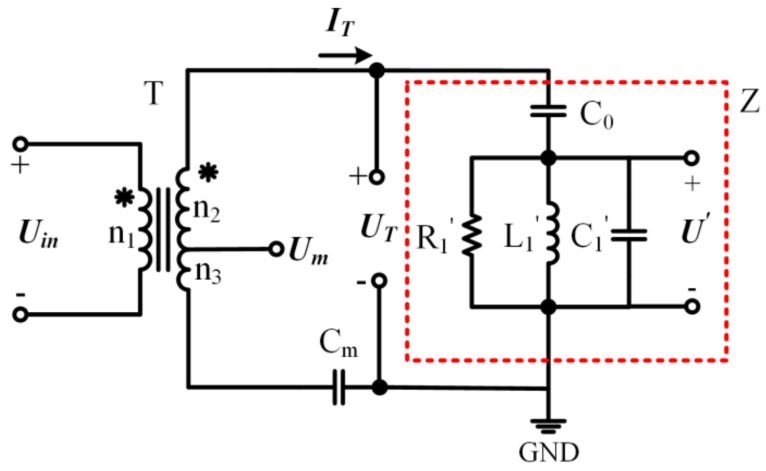
Transformer ratio-arm bridge.

The partial voltage $U^{\prime }$ is an equivalent voltage that cannot be measured directly. Therefore, a transformer ratio-arm bridge [23] is applied to detect $U^{\prime },$ as shown in Figure 4.

The transformer ratio-arm bridge consists of a transformer, a matching capacitance, and a piezoelectric element. Where T is a transformer with a tap. $U_{m}$ is the voltage of transformer tap. $C_{m}$ is the matching capacitance, which is set to as the following formula.

(10) $$C_{m}=\frac{n_{2}+n_{3}}{n_{3}}C_{0}$$

where $n_{1}$, $n_{2},$ and $n_{3}$ are the turns of the three windings of the transformer. The impedance of the matching capacitor $C_{m}$ is negligible compared to the impedance of the piezoelectric element because of the relatively larger ratio between $n_{2}$ and $n_{3}$. Therefore, the voltage of $C_{m}$ can be ignored when $U_{T}$ is calculated, which can be expressed as the following formula.

(11) $$U_{T}=\frac{n_{2}+n_{3}}{n_{1}}U_{in}=\frac{I_{T}}{j\omega C_{0}}+U^{\prime }$$

where $U_{in}$ is the input voltage of the transformer, while $n_{1}$, $n_{2},$ and $n_{3}$ are the turns of the three windings of the transformer. Then, $U_{m}$ can be expressed as the equation below.

(12) $$U_{m}=\frac{n_{3}}{n_{2}+n_{3}}U_{T}-\frac{I_{T}}{j\omega C_{m}}$$

Substitute Equation (10) and Equation (11) into Equation (12), the relationship between $U_{m}$ and $U^{\prime }$ can be described as the equation below.

(13) $$U^{\prime }=\frac{n_{2}+n_{3}}{n_{3}}\;U_{m}$$

Therefore, the voltage of transformer tap $U_{m}$ can characterize the SVA. Meanwhile, the phase difference θ can be obtained by the phase discriminator based on $U_{m}$ and $I_{T}$.

### 3.2. Control Structure

**Figure 5:**
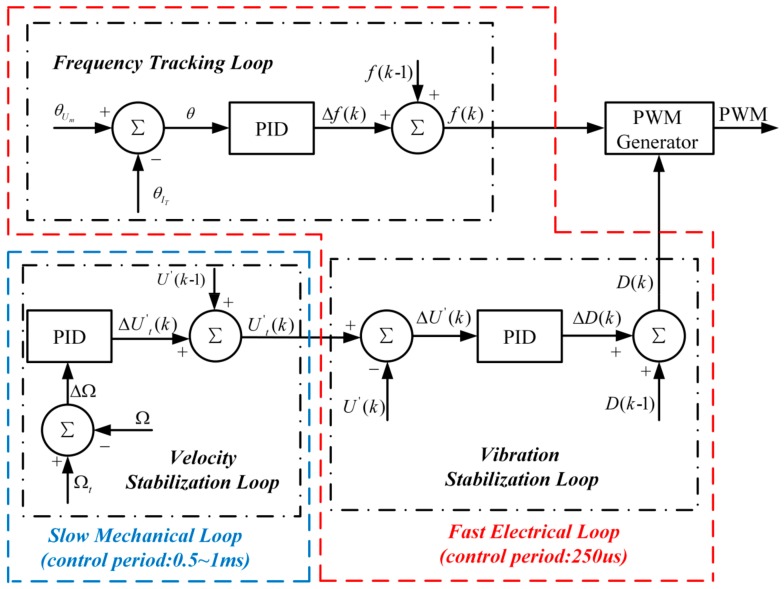
Control structure of proposed scheme.

In our research, the frequency of PWM waves is adjusted until θ arrives at 0°, so that the TWUSM operate at $f_{p}$. Meanwhile, the duty of PWM waves is adjusted to achieve the target SVA, and further control the velocity of the TWUSM. Therefore, three PID controllers are used to implement the proposed scheme, as shown in Figure 5. The sampling interval of the velocity Δt ranges from 0.5 ms to 1 ms when the velocity is 60–120 rpm. Meanwhile, the phase and the SVA are detected in real time by the circuit. In the proposed scheme, the input of velocity stabilization loop is velocity and the target SVA is output. Although the velocity stabilization loop is a slow mechanical loop due to the sampling interval of the velocity, the linear relationship between the velocity and the SVA could make the mechanical loop obtain the target velocity efficiently, according to the SVA. The vibration stabilization loop and the frequency tracking loop are the electrical loop, whose control period is set to 250 us in consideration of the response of the TWUSM. Therefore, the fast electrical loop could provide the target SVA in time.

In the frequency tracking loop, the PID controller can be expressed by the equation below.

(14) $$\Delta f\left(k\right)=f\left(k\right)-f\left(k-1\right)=P_{f}\theta \left(k\right)+I_{f}\overset{k}{\underset{j=0}{\sum }}\theta \left(j\right)+D_{f}\left[\theta \left(k\right)-\theta \left(k-1\right)\right]$$

where $P_{f}$, $I_{f},$ and $D_{f}$ are the proportional, integral, and differential coefficients, respectively. $\Delta f\left(k\right)$ is the increment of the driving frequency.

In the velocity stabilization loop, the velocity error, and ΔΩ is the velocity difference between measured velocity Ω and target velocity $\Omega_{t}$. The PID controller adjusts the target stator vibration amplitude $U_{t}\prime \left(k\right)$ to keep ΔΩ at 0. The relationship between the increment stator vibration amplitude $\Delta U_{t}\prime \left(k\right)$ and ΔΩ is expressed by the following equation.

(15) $$\Delta U_{t}\prime \left(k\right)=U_{t}\prime \left(k\right)-U_{t}\prime \left(k-1\right)=P_{U^{\prime }}\Delta \Omega \left(k\right)+I_{U^{\prime }}\overset{k}{\underset{j=0}{\sum }}\Delta \Omega \left(k\right)+D_{U^{\prime }}\left[\Delta \Omega \left(k\right)-\Delta \Omega \left(k-1\right)\right]$$

where $P_{U^{\prime }}$, $I_{U^{\prime }}$, and $D_{U^{\prime }}$ are the proportional, integral, and differential coefficient, respectively.

The duty $D\left(k\right)$ of PWM waves is adjusted by the PID controller to make the actual stator vibration amplitude $U^{\prime }\left(k\right)$ reach $U_{t}\prime \left(k\right)$ in the vibration stabilization loop. $D\left(k\right)$ can be calculated by the equation below.

(16) $$\Delta D\left(k\right)=D\left(k\right)-D\left(k-1\right)=P_{D}\Delta U_{t}\prime \left(k\right)+I_{D}\overset{k}{\underset{j=0}{\sum }}\Delta U_{t}\prime \left(j\right)+D_{D}\left[\Delta U_{t}\prime \left(k\right)-\Delta U_{t}\prime \left(k-1\right)\right]$$

where $P_{D}$, $I_{D},$ and $D_{D}$ are the proportional, integral, and differential coefficient, respectively.

## 4. Experimental Results

### 4.1. Experimental Setup

**Figure 6:**
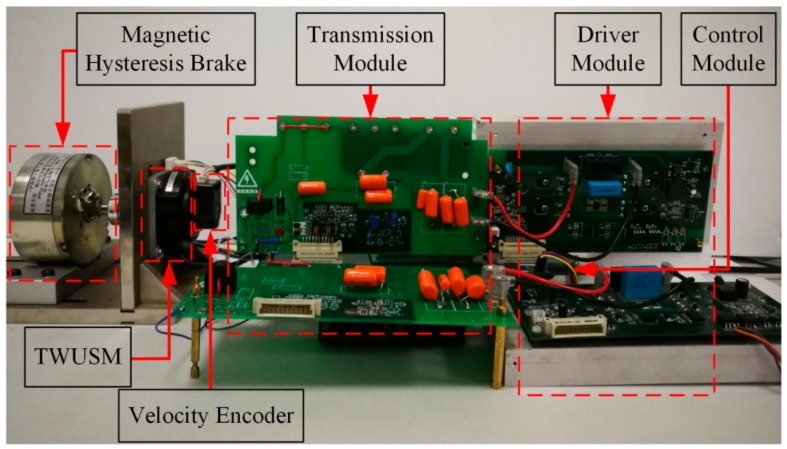
Experimental setup.

In this section, a commercial Shinsei USR60 (Shinsei, Tokyo, Japan) motor is used to verify the signal detection and the merits of a proposed scheme. Table 1 lists the main parameters of the TWUSM. A magnetic hysteresis brake provides the load torque in the experiment. The experimental setup for the TWUSM is shown in Figure 6. The input power of the experimental setup is supported by a DC power supply.

### 4.2. Verification of the Proposed Scheme

**Figure 7:**
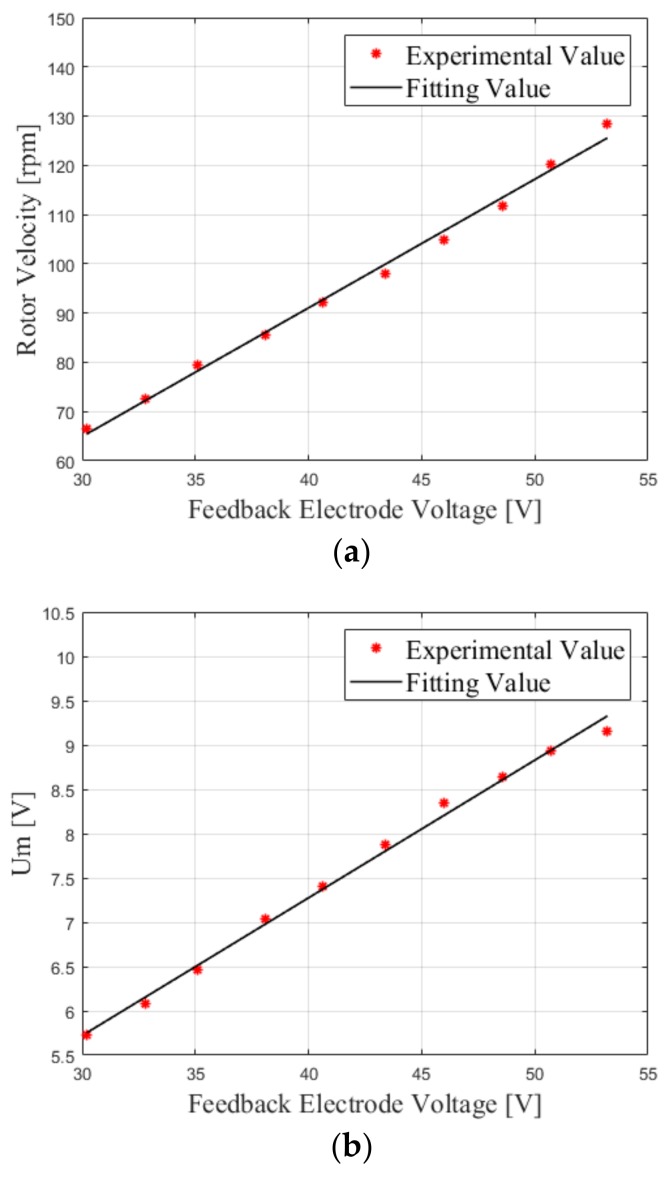
(**a**) Rotor velocity-feedback electrode voltage (no load) and (**b**) $U_{m}$-feedback electrode voltage (no load).

First, the correctness of the proposed scheme is verified. In the TWUSM, a feedback electrode is used to detect the SVA. When the stator vibrates, the feedback electrode generates a voltage that is proportional to the SVA, according to the positive piezoelectric effect [1]. The velocity of the TWUSM and the voltage of transformer tap $U_{m}$ are measured without load at different feedback electrode voltages. According to actual conditions of works, the velocity of the TWUSM ranges from 60–130 rpm. Figure 7a shows that the rotor velocity is almost linear to the SVA, being analyzed in Section 2.1. As shown in Figure 7b, $U_{m}$ has a linear relationship with the feedback electrode voltage, which verifies that the method proposed in Section 2.2 and Section 3.1 can detect the SVA without additional sensors.

**Figure 8:**
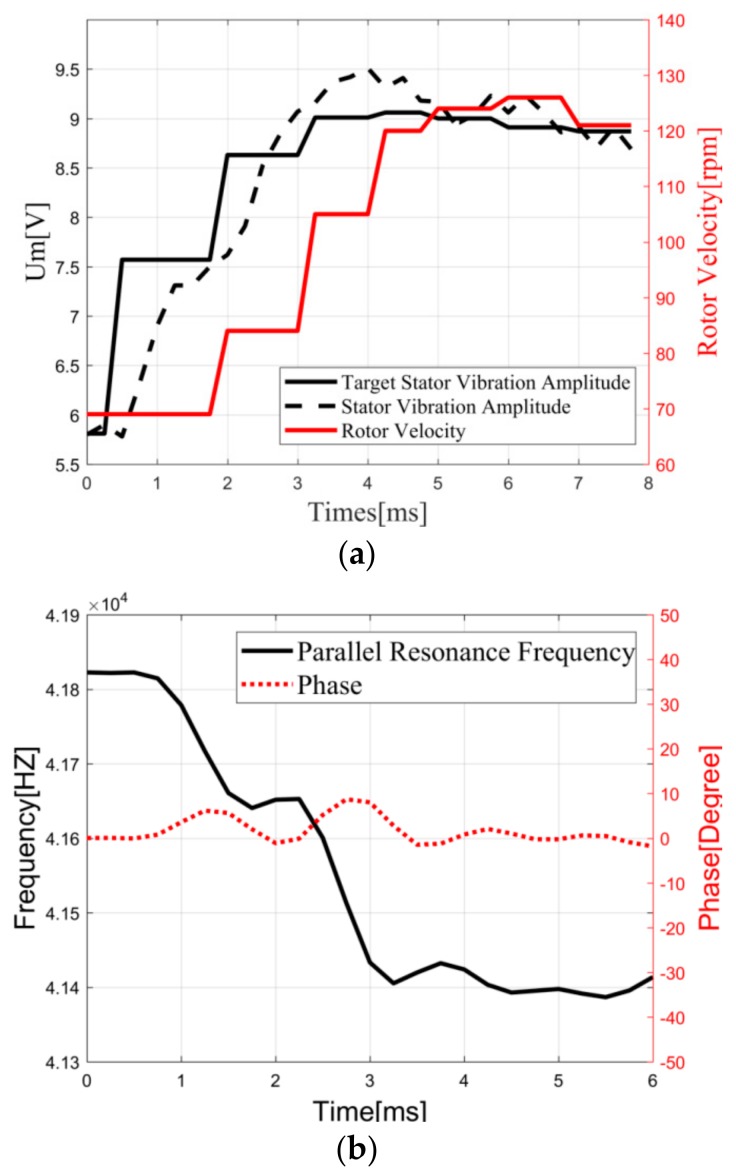
(**a**) Velocity and vibration stabilization process (no load) and (**b**) frequency tracking process (no load).

In order to verify the performance of three PID controllers, the phase difference, the SVA voltage, and the actual rotor velocity are measured as the rotor velocity increases from 70 rpm to 120 rpm without load. As shown in Figure 8a, target $U_{m}$ increases gradually from about 6 V to 9 V with the aid of the velocity stabilization loop. Then, under the action of the vibration stabilization loop, $U_{m}$ is gradually increased, so that the rotor velocity reaches the target value. The experimental results are consistent with the design of the velocity stabilization loop and the vibration stabilization loop in Section 3.2. As shown in Figure 8b, $f_{p}$ could be changed when the operating state of the TWUSM changes. Then, the phase difference θ is drifted from 0° due to the $f_{p}$ shift. However, the driving frequency is quickly adjusted to $f_{p}$ and θ return to 0° within 1 ms under the action of the frequency tracking loop. The phase error is within ±2° when the system is stable. The change of the phase indicates that the frequency tracking loop designed in Section 3.2 has been implemented when the $f_{p}$ shifts. This experiment shows that three controllers could realize the velocity control and $f_{p}$ tracking.

### 4.3. Merit of the Proposed Scheme

In order to verify the merit of the proposed scheme in terms of the dynamic performance, we design comparison experiments between the VCBVF and FBVC with 0 Nm, 0.2 Nm, 0.4 Nm, and 0.6 Nm, respectively. In addition, the velocity of the TWUSM increases from 70 rpm to 120 rpm.

**Figure 9:**
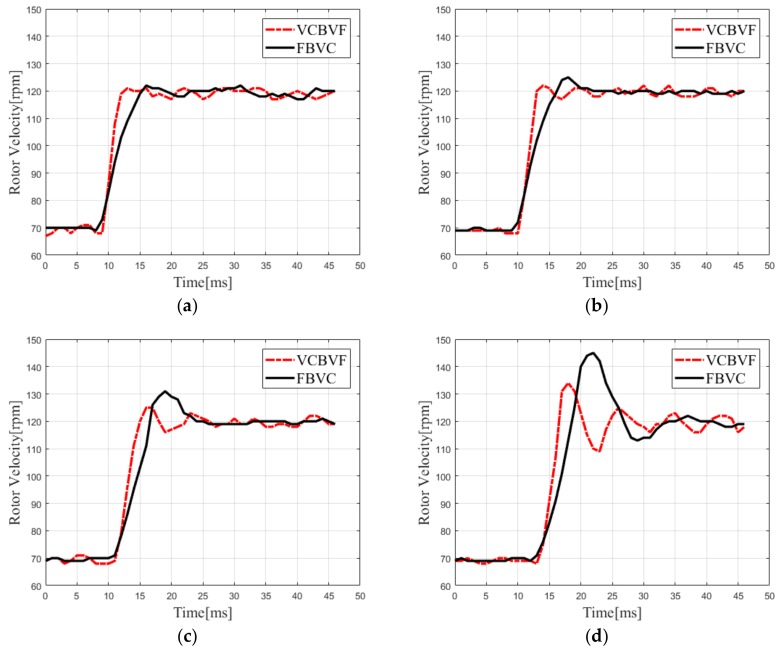
Rotor velocity response with different loads (70–120 rpm), (**a**) 0 Nm, (**b**) 0.2 Nm, (**c**) 0.4 Nm, and (**d**) 0.6 Nm.

The rotor velocity response of the TWUSM with different loads is shown in Figure 9. From the experimental results, the dynamic performance of the TWUSM is shown in Table 2. The rise time of the rotor velocity in the VCBVF is about 3.0–4.0 ms, which is less than that in the FBVC. Moreover, the VCBVF scheme has a smaller rotor velocity overshoot than the FBVC scheme. Therefore, the velocity response of the VCBVF is faster due to the control method rather than the PID parameters.

In addition, when the load torque for the TWUSM changes from 0 Nm to 0.6 Nm, the overshoot of the VCBVF scheme increases from 2.0% to 22.0%, while the overshoot of the FBVC scheme increases from 4.0% to 40.0%. It proves that the VCBVF scheme has better load adapting ability than the FBVC scheme. Due to the linear relationship between the velocity and the SVA, the proposed scheme could improve the dynamic performance of the TWUSM, which is analyzed in Section 3.2.

**Figure 10:**
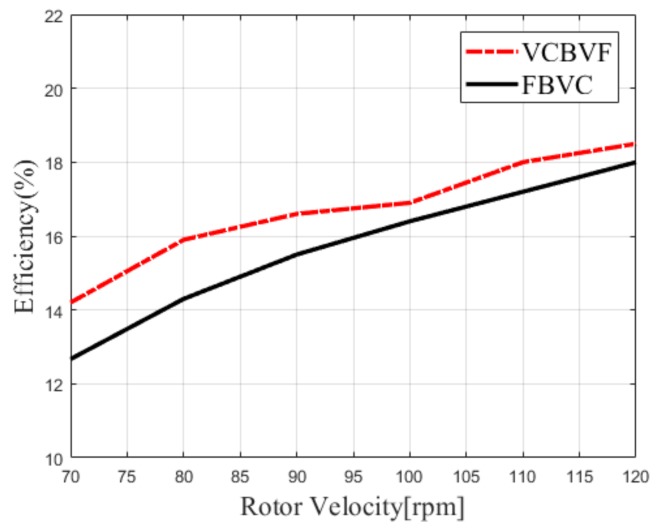
Efficiency of the TWUSM controlled by two schemes (Load torque T_L_ = 0.3 Nm).

The efficiency η is an important indicator of the scheme and is the ratio of the mechanical output power of system $P_{out}$ to the electrical input power of system $P_{in}$. The efficiency η can be described by the equation below.

(17) $$\eta =\frac{P_{out}}{P_{in}}\times 100\%=\frac{\pi \Omega \cdot T_{L}}{30U_{DC\_in}\cdot I_{DC\_in}}\times 100\%$$

where Ω is the rotor velocity with a unit of rpm, $T_{L}$ is load torque, $U_{DC\_in}$ is the input DC voltage, and $I_{DC\_in}$ is the input DC current. As shown in Figure 10, the efficiency of the VCBVF scheme is superior to that of the FBVC scheme in the experiment. When driving frequency drifts from $f_{p}$, the electromechanical coupling efficiency decreases significantly and the loss of the circuit increases slightly. The former dominates the decrease of efficiency [15]. Therefore, the proposed scheme can improve the efficiency of the TWUSM due to the frequency tracking loop.

## 5. Conclusions

In conclusion, this paper proposes the VCBVF of the TWUSM. The SVA and $f_{p}$ are detected by a transformer ratio-arm bridge rather than a feedback electrode. Then, the proposed scheme achieves the mechanical loop and the electrical loop. Although the mechanical loop may be slow, the mechanical loop gets the target velocity efficiently due to the linear relationship between the velocity and the SVA. Then, the electrical loop can quickly achieve the target SVA. The results show that the dynamic performance of the TWUSM in the proposed scheme is better than in the traditional scheme. In addition, the efficiency of the proposed scheme is higher than the traditional scheme. The proposed scheme could be very useful for the control of the TWUSM, especially under conditions with a wide range of velocity and load, such as the robot control system.

## Figures and Tables

**Table 1:** Main parameters of the Shinsei USR60.

Driving Frequency	40–45 kHz
Maximum Driving Voltage	130 Vrms
Maximum Torque	1.0 Nm
Rated Output	5.0 W
Maximum Velocity	150 rpm

**Table 2:** Dynamic performance of the TWUSM.

	VCBVF		FBVC	
Load (Nm)	Rise Time (ms)	Overshoot (%)	Rise Time (ms)	Overshoot (%)
0	3.0	2.0	6.0	4.0
0.2	3.0	4.0	6.0	10.0
0.4	4.0	10.0	6.0	18.0
0.6	4.0	22.0	7.0	40.0
